# Comparison of Ammonia Emission Estimation between Passive Sampler and Chamber System in Paddy Soil after Fertilizer Application

**DOI:** 10.3390/ijerph17176387

**Published:** 2020-09-02

**Authors:** Min-Suk Kim, Namin Koo, Seunghun Hyun, Jeong-Gyu Kim

**Affiliations:** 1 O-Jeong Resilience Institute, Korea University, Seoul 02841, Korea; adoniss86@korea.ac.kr (M.-S.K.); soilhyun@korea.ac.kr (S.H.); 2 Forest Conservation Department, National Institute of Forest Science, Seoul 02455, Korea; koosor@korea.kr; 3 Division of Environmental Science and Ecological Engineering, College of Life Science and Biotechnology, Korea University, Seoul 02841, Korea

**Keywords:** ammonia inventory, dry deposition, emission flux, fertilizer spreading, rice transplanting, wind rose diagram

## Abstract

Ammonia (NH_3_) is an important precursor for particulate secondary aerosol formation. This study was conducted to evaluate the applicability of a passive sampler (PAS) for estimating the NH_3_ emission from chemical fertilizer application (85 kg-N·ha^−1^) at field scale and to compare the results with a chamber system for the calculation of NH_3_ emission flux at lab scale. The application of chemical fertilizer increased the ambient NH_3_ concentration from 7.11 to 16.87 μg·m^−3^. Also, the ambient NH_3_ concentration measured by the PAS was found to be highly influenced by not only the chemical fertilizer application but also the weather (temperature and rainfall). Wind rose diagram data can be useful for understanding the distribution of ambient NH_3_ concentration. In the case of a chamber with few environmental variables, NH_3_ was emitted very quickly in the early stages and gradually decreased, whereas it was delayed at intervals of about one week at the site. It was found that daily temperature range, atmospheric disturbance by wind and rainfall, changes in soil moisture, and the presence of a flooded water table were the main influencing factors. The PAS data and the chamber system data were observed to have significant differences in spatial-temporal scale. In order to reduce the gap, it seems to be necessary to further develop a chamber system, in order to improve the precision of field analysis and to strengthen the connection between experimental results.

## 1. Introduction

Particulate matter (PM) is a major source of toxicity for human health worldwide and is a bigger problem in Asian countries than in Europe and the United Sstates [1]. In 2013, the International Agency for Research on Cancer (IARC) classified fine PM (<2.5 μm) as a group 1 carcinogen that is recognized as causing cancer in humans and the World Health Organization (WHO) reported that more than 7 million people died earlier than life expectancy due to fine PM in 2014 [2]. In Korea, the average concentration of fine PM was 32.0 μg·m^−3^ in 2015, which was the worst value among 25 Organization for Economic Cooperation and Development (OECD) countries [3]. Although the government has implemented various countermeasures to improve the air quality, there are still frequent cases of high concentrations of PM, so the impact of the efforts to improve the air quality felt by the public has been insufficient [4].

PM can be emitted directly into the air or formed through conversion of gaseous state to particulate state, and the chemical constituents that make up PM (called precursors) are various including organic carbon, nitrogen oxides (NOx), sulfur oxides (SOx), ammonia (NH_3_), and trace metals [5,6]. Although the exact mechanism of occurrence of urban fine PM is still uncertain, NOx and volatile organic compounds (VOCs) emitted from vehicles seems to be the major causes [7]. On the other hand, in the case of rural areas, the ambient PM is increased by the emission of PM directly from livestock facilities or agricultural machines and by the emission of precursors of particulate matter such as NH_3_ from livestock manure and nitrogen-based chemical fertilizers [8].

Among several precursors, NH_3_ is the only basic gas in ambient air that reacts easily with acidic gases such as SOx and NOx to form salts, and thus, emission management and related information are relatively insufficient for other substances [9]. According to the NH_3_ inventory in Korea, in 2015, more than 77% of the emitted NH_3_ originated from the agricultural sector, with emissions resulting from manure management and fertilizer application [10]. However, without considering the seasonal change and cropping system, the emission is simply calculated by multiplying the emission factor (NH_3_—kg·unit^−1^) and activity (unit·year^−1^) based on statistical data and established literature [9]. Therefore, in order to accurately quantify the amount of NH_3_ emission, monitoring of the ambient NH_3_ concentration and its deposition is necessary.

A passive sampler (PAS) is very suitable tool for long-term measurement in a large area and has the advantages of being easy to handle and cost efficient [11]. In the USA, the National Atmospheric Deposition Program (NADP) and the Ammonia Monitoring Network (AMoN) have continuously monitored NH_3_ concentrations in the long-term scale, and they reported that the concentration of NH_3_ and the precipitation of ammonium (NH_4_^+^) shows increasing trends over a large area of the USA [12]. Zbieranowski and Aherne [13] utilized a PAS to evaluate the spatial and temporal variability in ambient NH_3_ in Canada over three years and reported that agricultural statistical data are well correlated between NH_3_ concentration and livestock (pig and cattle) heads. Rabaud et al. [14] also used a PAS for the determination of airborne NH_3_ near a large scale livestock facility. Zhang et al. [15] and Hojito et al. [16] estimated the annual nitrogen (N) deposition in grassland and monitored the vertical distribution of NH_3_ near an intensive dairy farming area. However, relatively few studies have conducted NH_3_ monitoring after fertilizer application using PAS [17], and domestic research progress is not significantly different.

Therefore, the purpose of this study was to confirm the applicability of a PAS for monitoring the tendency of NH_3_ emission during fertilizer treatment in paddy soils in field conditions and to compare the results of NH_3_ emission calculated through a chamber system in the laboratory.

## 2. Materials and Methods

### 2.1. Site Description and Fertilizer Application

**Figure 1:**
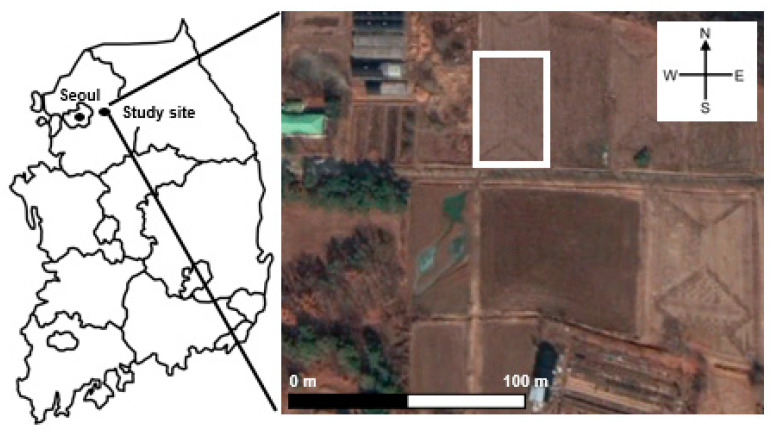
Satellite view (Google Earth Pro, 2020) of the study site (outlined in white) and neighboring fields.

The agricultural paddy soil (1400 m^2^) is located at Namyangju city, Gyeonggi province, Korea. (37°35′00.8″ N, 127°14′17.8″ E) and has been farming rice for several years (Figure 1). The pH, clay content, bulk density and soil organic carbon of the field paddy soil were 6.2, 22%, 1.2 g·cm^−3^ and 3.0%, respectively [18] with the soil analysis was conducted according to the guidelines of National Institute of Agricultural Science and Technology (NIAST) [19]. The paddy soil was left unattended during the winter season and tillage was conducted on 23 April 2020. After two weeks (7 May 2020), commercial composite fertilizer (25% nitrogen content) was applied with the input ration of 85 kg-N·ha^−1^ and then, the paddy soil was mixed and irrigated. After two weeks of aging (21 May 2020), the seedlings of rice were transplanted during submerged conditions. Before the fertilizer application, soil samples were collected for the chamber experiment in the laboratory. The collected soil samples were air-dried, passed through a 4 mm sieve, and stored in a polyethylene bottle until used in the chamber experiment.

### 2.2. On-Site Passive Samper Experiement

The commercial Radiello^®^ passive samplers (PAS) (Radiello, Pavia, Italy) was selected for the field monitoring of NH_3_ concentrations. Each sampler included a blue diffusive body (RAD1201), cartridge adsorbents (RAD168), and vertical adapter (RAD122), which were purchased through Sigma Aldrich. Each sampler was put in a rain shelter made of stainless steel that was placed approximately 2 m above ground. A total of 17 shelters were installed in a zigzag formation to cover all of the experimental area. After one week of exposure, each diffusive body and cartridge was replaced with a new one every week, and the experiment was performed during six weeks from 23 April 2020 to 4 June 2020. By placing two repetitions in one shelter, a total of 204 cartridge samples were collected. Cartridges collected one week after exposure were immediately sealed and brought to the laboratory. The NH_3_ adsorbed on the cartridge was extracted with 10 mL of deionized water and a vortex, and the NH_4_^+^ concentration in the extractant was quantified by indophenol blue colorimetric method using a UV-VIS spectrophotometer (Optizen POP, Mecasys Co. Ltd., Daejeon, Korea). A blank value did not exceed 0.040 absorbance units. The limit of detection (LOD), limit of quantification (LOQ) were 0.036 and 0.119 NH_4_-N mg·L^−1^, respectively. The ambient NH_3_ (C_N_, μg·m^−3^) concentration was calculated as follows:

C_N_ = 0.944 × *m* × 1,000,000/235/*t* (1)

where *m* is the mass of NH_4_^+^ (μg) in the cartridge, *t* is the exposure time (minutes) and 0.944 is the factor to convert NH_4_^+^ to NH_3_. The value of 235 refers to the sampling rate (Q_298_, mL·min^−1^) at 298K and 1013 hPa. For quality assurance and quality control, parallel duplicate samples, field blanks and travel blanks were performed [20]. The LOD and uncertainty values were 1 μg·m^−3^ for 24 h exposure and 6.5% at 2σ, respectively. In order to confirm the effect of the weather, data (temperature, rainfall and wind) were collected from nearby a national automatic weather system (AWS) in Korea.

### 2.3. Chamber Experiement in Laboratory

Since a PAS collects NH_3_ that is deposited, a dynamic chamber-capture system (DCS) was used to quantify the amount of NH_3_ emitted from fertilizer after soil application [21]. Briefly, after application of fertilizer to the soil at the same ratio as the treated site (85 kg-N·ha^−1^), the chamber was sealed, and all air was passed through a boric acid solution (5%) in the bubbler to capture the entire amount of NH_3_ using a vacuum pump (15 L per min). Captured NH_3_ was immediately quantified by indophenol blue colorimetric method.

First, 10 cm of soil was placed in the chamber and sealed without fertilizer application. At this time, the moisture content of soil was standardized by using measurement of the moisture content of the field soil at the date of installation of the PAS cartridge (23 April 2020). In order to ensure constant moisture content of the soil, moisture was supplied according to the weight every day. The NH_3_ emitted from the soil was continuously captured for two weeks using a boric acid bubbler, and the temperature was adjusted daily according to the observed average daily temperature by AWS. After two weeks, the chamber was opened, fertilizer was added, and irrigation was performed until the soil was saturated, to the same conditions as recorded in the field (7 May 2020). After closing the chamber, the NH_3_ was captured and quantified in the same manner as in the method during the previous four weeks. However, the chamber was unable to simulate the transplanting treatment in the field, so NH_3_ emission was continuously measured for four weeks after the fertilizer treatment. In addition, in order to compare the effect of irrigation on NH_3_ volatilization, an additional treatment that did not include irrigation after the fertilizer application was also performed.

The bubbler containing the boric acid solution was replaced with a new one every 24 h after the start of the experiment, and the NH_3_ emitted from the soil was quantified once a day. After the quantification of captured NH_3_, the NH_3_ emission flux (mg·m^−2^·h^−1^) was calculated considering the surface area of the chamber (m^−2^). The process and results of quality assurance and quality control were confirmed through a previous study [21] and all of the chamber experiments were performed in three replicates.

## 3. Results and Discussion

### 3.1. Site Weather Information during the Experiment

**Figure 2:**
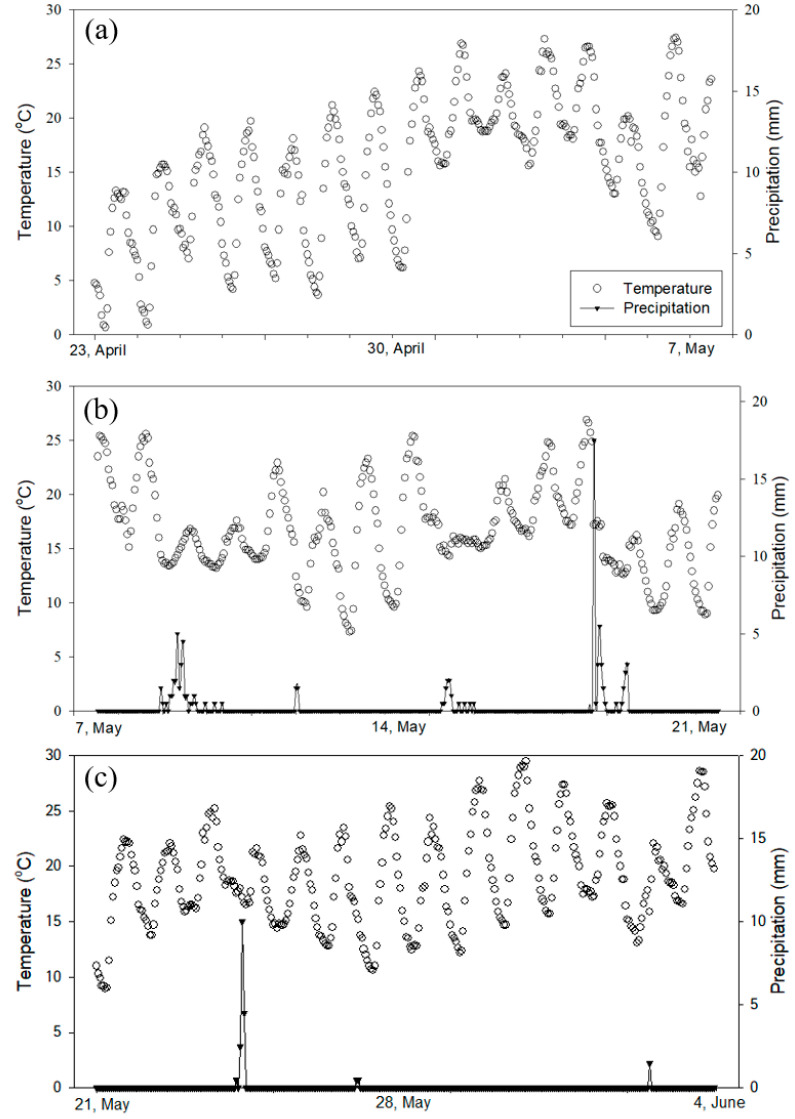
Temperature (left y-axis) and precipitation (right y axis) in the study site during April 23–June 4. (**a**) After tillage (April 23–May 7); (**b**) After chemical fertilizer application and irrigation (May 7–May 21); (**c**) After transplanting rice seedlings (May 21–June 4).

The average, minimum, and maximum temperatures during the six-week field study period were 17.21 °C, 11.47 °C, and 23.62 °C, respectively. The average, minimum, and maximum temperatures for the two weeks after tillage were 15.75 °C, 8.77 °C, and 22.74 °C, respectively (Figure 2a). The average, minimum, and maximum temperatures for the two weeks after chemical fertilizer application and irrigation were 16.77 °C, 11.81 °C, and 22.55 °C, respectively (Figure 2b). The average, minimum, and maximum temperatures for the two weeks after transplanting the rice seedlings were 19.28 °C, 13.72 °C, and 25.77 °C, respectively (Figure 2c). During the first two weeks after tillage, there was no effective rainfall (Figure 2a). In the two weeks after the chemical fertilizer application and irrigation, a total of 80.5 mm of rain fell (Figure 2b). After transplanting the rice seedlings, it rained 20.0 mm until the end of the experiment (Figure 2c). The season in the study site was spring, and as the experiment progressed, the temperature showed an increasing trend. During rainy conditions, the average temperature was relatively low. In the second period (Figure 2b), it rained the most, and the average temperature was relatively low.

**Figure 3:**
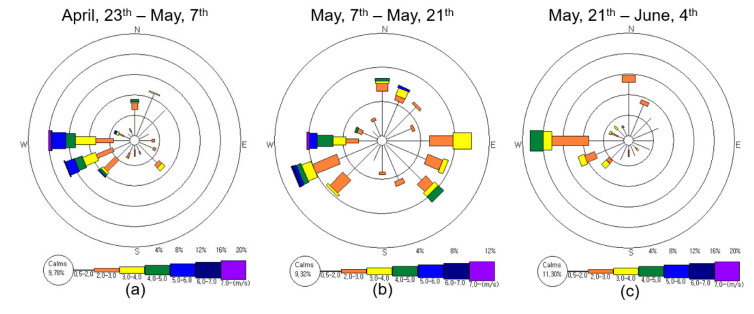
Wind rose diagrams of the study site during April 23–June 4. Calm wind refers to when the wind speed is lower than 0.5 m·s^−1^. (**a**) After tillage (April 23–May 7); (**b**) After chemical fertilizer application and irrigation (May 7–May 21); (**c**) After transplanting rice seedlings (May 21–June 4).

The average wind speed during the six-week field study period was 1.68 m·s^−1^. The average wind speed according to the three aforementioned periods discussed for temperature, was 1.90 m·s^−1^, 1.69 m·s^−1^, and 1.44 m·s^−1^, respectively (Figure 3). The proportion of calms wind (under 0.5 m·s^−1^) during each period was 9.78%, 9.32%, and 11.30%, respectively. After transplanting the rice seedlings, the average wind speed was the lowest and the most silent period (Figure 3). The main wind direction of the six-week period, not including periods with calm wind (10.4%) was (in the order of highest to lowest) west-south-west (14.1%), west (10.7%), and north-north-east (9.4%). This trend of wind direction was similar after tillage (Figure 3a) and after transplanting the rice seedlings (Figure 3c). During the two weeks of heavy rain (Figure 3b), various wind directions were observed due to atmospheric disturbance and instability.

### 3.2. Ambient NH_3_ Concentration in the Study Site

**Figure 4:**
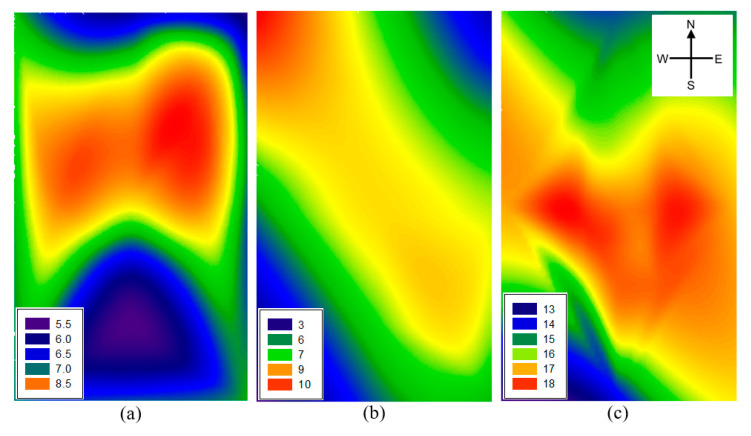
Changes in the spatial distribution of ammonia (NH_3_) concentration (μg·m^−3^) in the study site. (**a**) After tillage (April 23–May 7); (**b**) After chemical fertilizer application and irrigation (May 7–May 21); (**c**) After transplanting rice seedlings (May 21–June 4).

The average concentration, standard deviation, and minimum-maximum range of ambient NH_3_ during the six-week study period was 11.29 μg·m^−3^, 5.02 μg·m^−3^, and 5.47–19.96 μg·m^−3^, respectively. Divided into two-week periods according to the order of the experiment, the concentration of ambient NH_3_ gradually increased to 7.11, 7.25 and 16.87 μg·m^−3^, respectively (Figure 4). High concentrations of NH_3_ were distributed around the center of the target study site during all experiments. The southern part of study site is adjacent to the road, resulting in generally low concentrations of NH_3_ due to the distance from the nearby farmland. It has been previously shown that the main sources of atmospheric NH_3_ were animal manure and N-based fertilizers [22]. Dry deposition of NH_3_ is a one of the major removal mechanisms from ambient air to soil surfaces, and a PAS can adsorb and capture falling NH_3_ by diffusion [22,23]. Since the area around the study site is also agricultural land, NH_3_ from the surrounding area may have been captured in the PASs of our study site. However, as NH_3_ concentration is high in the central part where the PASs were installed and decreased toward the boundary of the periphery, it has been supposed that the NH_3_ emitted from our research site was mainly collected and that the results of spatial distribution were observed. During the two-week capture period (April 23–May 7), there was no compost and fertilizer use in the study soil or any surrounding soils, and the deposition and capture distribution of NH_3_ was concentrated in the center of study site, as shown in Figure 4a. Despite the fact that fertilizer was not applied, a significant amount of NH_3_ was emitted (in the 5.47–8.43 μg·m^−3^ range), and the reason for this could be found in the tillage practice. During the cold winter, the soil that was not cultivated and that was left unattended begins the next farming cycle with tillage in the spring season. Tillage loosen the top layer of soil, enhances organic nitrogen mineralization, and reduces the resistance to NH_3_ diffusion by top soil, resulting in the production of ammonium (NH_4_^+^) and the emission of NH_3_ [24].

After the chemical fertilizer application, the average concentration of ambient NH_3_ during the two weeks was 7.25 μg·m^−3^ (Figure 4b). Hayashi et al. [25] reported that NH_3_ volatilization began on the day after fertilizer application (63.6 kg N·ha^−1^) in an upland field where there was nothing that interfered with the volatilization of NH_3_. On the contrary, the transformation and behavior of nitrogen from fertilizer is unique in paddy soil due to the effect of the layer of the free water table. A flooded water table interferes with the supply of oxygen to the soil, resulting in the creation of redox conditions and enhancing runoff, seepage, and the leaching of nitrogen [26]. Also, rainfall can reduce NH_3_ volatilization in soil and enhance wet deposition in the atmosphere, causing a decrease in ambient NH_3_ concentrations [27,28]. In this study, irrigation was carried out immediately after the fertilizer application, and after that, a total of 80.5 mm of rain fell on four rainfall events in two weeks. For this reason, despite the application of nitrogen chemical fertilizer, NH_3_ emission and deposition did not increase when compared to after tillage (Figure 4a).

A significant increase in ambient NH_3_ concentrations was observed after transplanting the rice seedlings (Figure 4c). It is known that NH_3_ volatilization is increased in paddy soils as the NH_4_^+^ concentration in floodwater increases and the flooded water table decreases [29]. Also, an increase in temperature is an intuitive factor for increasing ambient NH_3_ concentrations. The increases in soil temperature promote soil microbial activity and increase the NH_3_/NH_4_^+^ ratio in the liquid phase of the flooded water table, resulting in enhancement of NH_3_ volatilization [30,31]. During the transplanting on May 21, the paddy soil and water table were disturbed, and there was only one rainfall event (20.0 mm) after planting. Therefore, as time went by, the amount of NH_4_^+^ in the flooded water was increased and at the same time the flooded water table was decreased due to evaporation, resulting in the increases in ambient NH_3_ concentrations. Rice has been known to act as an absorber for NH_3_ in the atmosphere and at the same time serves as an emitter [32]. However, in this study, it is considered that this effect was negligible because rice seedlings are small in size only two weeks after transplantation.

The effect of rainfall on ambient NH_3_ distribution could be confirmed by comparing Figure 3 and Figure 4. In Figure 3a,c, the wind direction is consistently common, which shows a high concentration of ambient NH_3_ in the center of the target study site (Figure 4a,c). In addition, the frequency of high wind speeds was not high, and the situation with little or no rainfall seems to have contributed to this result. Between May 7 and May 21, when the rain was relatively frequent, the direction and frequency of the wind was very diverse, resulting in a different trend of distribution of ambient NH_3_ concentrations compared to Figure 4a,c.

### 3.3. Long-Term Monitoring of NH_3_ Emission using the Chamber System

**Figure 5:**
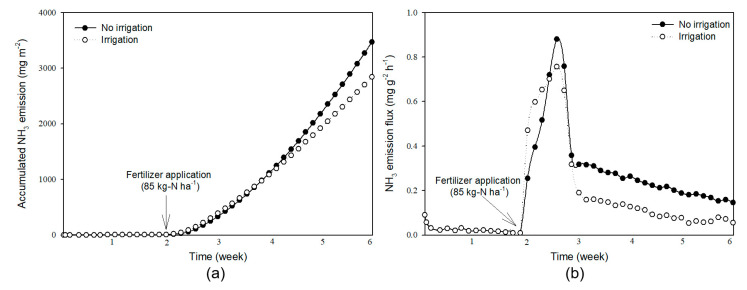
Changes in accumulated NH_3_ emission (**a**) and NH_3_ emission flux (**b**) when chemical fertilizer was applied to soil during a six-week period.

The amount of volatilized NH_3_ was captured and determined for six weeks (Figure 5) using a chamber system with a recovery rate of 98.2% [21]. The total amount of NH_3_ emitted from soil during the two weeks after tillage was 52.02 mg·m^−2^. After fertilizer application, the NH_3_ emission increased dramatically, and the accumulated emission was higher when irrigation was not performed. The total amount of NH_3_ emitted from soil during the six weeks was 2839.04 mg·m^−2^ and 3466.69 mg·m^−2^, respectively, with and without irrigation practice (Figure 5a). The cumulative emissions continued to increase after fertilizer application, while the NH_3_ emission flux per unit hour was highest in the first week immediately after fertilizer application (Figure 5b). In the early stage immediately after fertilizer application, the emission flux was faster when irrigation was performed, but the maximum emission flux was higher when irrigation was not performed (0.88 mg·g^−2^·h^−1^). From the second week after fertilizer application, the emission flux gradually decreased, but it was still higher than before the fertilizer application. Additionally, the emission flux was always higher when the irrigation was not performed than when irrigation was performed throughout the entirely of the chamber experiment.

In general, there are three types of nitrogen fertilizers that can be applied to agricultural soil: solid compost from manure, sludge, or food waste; solid chemical fertilizer; and liquid fertilizer from manure [33]. In order for NH_3_ to be volatilized into the atmosphere from the solid types of fertilizer, nitrogen in the fertilizer should be primarily dissolved into the solution in the form of NH_3_ or NH_4_^+^ [26]. Most nitrogen in chemical fertilizers is in the form of NH_4_^+^ or NO_3_^−^, so it is easy to volatilize after dissolution. However, nitrogen in organic waste resources is often present in the form of organic matter, which takes more time to mineralize until volatilization with NH_3_. Gao et al. [34] observed that the mineralization of nitrogen and the resulting increase in NH_3_ volatilization appeared after 6 days of incubation. DeLaune et al. [35] also reported that it took 10 to 14 days for the solid chemical to dissolve, resulting in lower NH_3_ emissions from poultry litter. In the case of liquid fertilizer and liquid manure or slurry, a large amount of nitrogen is present in the form of total ammoniacal nitrogen (TAN, NH_3_ + NH_4_^+^), so it is relatively easy to emit to the atmosphere [36]. Kim et al. [21], who considered the NH_3_ emission from livestock liquid fertilizer (the ratio of TAN to TN is 86%), determined that the emission flux (mg·m^−2^·h^−1^) was the highest immediately after spreading, and rapidly decreased for 24 h, after which it showed a very low value.

**Figure 6:**
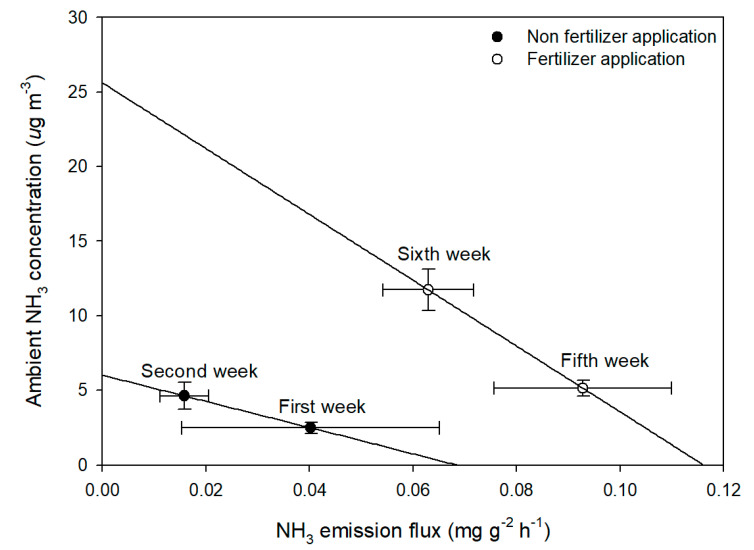
Relationship between NH_3_ emission flux by chamber experiment at lab scale and ambient NH_3_ concentration by passive sampler experiment at field scale.

To examine to what extent a PAS could reflect NH_3_ emission from soil, the relationship between on-site PAS results (y-axis) and lab-chamber results (x-axis) was confirmed excluding the results of the third and fourth weeks, which had been disturbed by rainfall (Figure 6). The PAS result corresponding to the y-axis was relatively small due to the variation in the concentration of NH_3_ in the cartridges captured from 17 shelters for a week, but the emission flux corresponding to the x-axis was determined in units of one day, resulting in a large deviation. Also, the deviation was very large in the first and third weeks because the emission flux was high after tillage (first week) and after the fertilizer application (third week), and decreased rapidly with time. At the second and sixth weeks, the emission flux from the chamber decreased compared to the first and fifth weeks, but the ambient NH_3_ concentration was higher at the field site. There might be several reasons for this trend. First, the set temperature in the chamber utilized the average daily temperature observed in the field, so the maximum daily temperature was not sufficiently reflected in the chamber. For example, average temperatures were 8.1 °C and 16.5 °C but the highest temperatures were 13.3 °C and 22.4 °C on April 23 and May 21, respectively. It has been shown that increases in surface temperature from high radiation levels from daylight have the effect of increasing atmospheric instability, resulting in the enhancement of NH_3_ volatilization [37]. Therefore, in the second and sixth weeks, when there were many days when the highest temperature was high, more NH_3_ was emitted from the soil, and it seems that the ambient concentration in the atmosphere increased. Another reason would be the effect of soil moisture. During the first and second weeks, while water was added and adjusted daily to maintain the initial soil moisture during the chamber experiment, rainfall was the only source of moisture in the field experiment. Increased soil moisture promotes soil microbial activity and TAN concentrations, thereby enhancing NH_3_ emission [38]. During the fifth and sixth weeks, a reduction of the flooded water table by evaporation caused high TAN concentrations in solution and thus high NH_3_ emission losses [39]. In addition, the 20.0 mm rainfall during fifth week also seems to have contributed to diluting the TAN in the flooded water table (Figure 2c) [40]. In addition to these factors, changes in wind speed, influences from nearby farmland, and the delay effect mentioned in the previous section may also have had impacts.

## 4. Conclusions

In this study, the applicability of a PAS to monitoring the tendency of NH_3_ emissions during fertilizer treatment in paddy field soil was evaluated and compared with the results of NH_3_ emission calculated through a chamber system in the laboratory. It was confirmed that a PAS could be utilized to demonstrate the distribution of ambient NH_3_ concentrations in agricultural land. Chamber systems also seem to be utilizable for estimating NH_3_ emission fluxes and trends. However, it seems that the NH_3_ volatilization into the atmosphere after fertilizer application was delayed by flooded water table conditions and rainfall of considerable intensity. Additionally, it was determined that wind rose diagram information is useful for interpreting the ambient NH_3_ concentration results. A chamber system that is capable of capturing and analyzing the entire amount of NH_3_ emitted from the soil should be developed in order to allow for accurate determination of NH_3_ emission. In order to allow for a more accurate understanding of experiment results produced in both field and laboratory settings, chamber systems and PASs should be used in tandem more often. Additionally, their combined usage could be especially important for strengthening the connection with weather data.
